# Albendazole is effective in controlling monogeneans in *Colossoma macropomum* (Serrasalmidae): therapeutic baths and their physiological and histopathological effects

**DOI:** 10.1590/S1984-29612024044

**Published:** 2024-08-26

**Authors:** Raimundo Rosemiro de Jesus Baia, Carliane Maria Guimarães Alves, Marcos Sidney Brito de Oliveira, Clara Brito Salomão, Abthyllane Amaral de Carvalho, Marcela Nunes Videira, Eliane Tie Oba Yoshioka, Marcos Tavares-Dias

**Affiliations:** 1 Programa de Pós-graduação em Biodiversidade Tropical – PPGBIO, Universidade Federal do Amapá – UNIFAP, Macapá, AP, Brasil; 2 Laboratório de Morfofisiologia e Sanidade Animal, Universidade do Estado do Amapá – UEAP, Macapá, AP, Brasil; 3 Embrapa Amapá, Macapá, AP, Brasil

**Keywords:** Anthelmintic, parasites, tambaqui, treatment, Anti-helmíntico, parasitos, tambaqui, tratamento

## Abstract

In aquaculture worldwide, most of the chemotherapeutic agents used for disease control and treatment are unregulated chemical products derived from agriculture. In this study, we investigated the efficacy of therapeutic baths with albendazole against the monogeneans *Anacanthorus spathulatus*, *Notozothecium janauachensis* and *Mymarothecium boegeri*, which infest the gills of *Colossoma macropomum*, and the hematological and histopathological effects of this anthelmintic agent on these fish. Albendazole at a concentration of 500 mg/L was used in three baths of 24 hours each, with intervals of 24 hours between these baths. Three replications of this treatment were used, and the control group consisted of water from the cultivation tank. Afterwards, hematological, histopathological and parasitological analyses were conducted. We found that the therapeutic baths with albendazole at 500 mg/L presented high efficacy (94.9%) against monogeneans de *C. macropomum* and caused few physiological or histopathological alterations. Therefore, baths with albendazole at 500 mg/L, as used in this strategy, can be recommended for controlling and treating infections by monogeneans in *C*. *macropomum*.

## Resumo

Na aquicultura mundial, a maioria dos quimioterápicos utilizados no controle e tratamento de doenças, são produtos químicos não regulamentados e oriundos da agricultura. Neste estudo, foi investigada a eficácia de banhos terapêuticos com albendazol contra monogeneas *Anacanthorus spathulatus*, *Notozothecium janauachensis* e *Mymarothecium boegeri* das brânquias de *Colossoma macropomum*, e os efeitos hematológicos e histopatológicos causados por esse anti-helmíntico nos peixes. Foram usados 500 mg/L de albendazol e um grupo controle (água do tanque de cultivo) com três banhos de 24 horas, mantendo intervalos de 24 horas entre cada banho, usando três repetições cada. Ao final dos banhos terapêuticos, foram realizadas análises hematológicas, histopatológicas e parasitológicas. Verificou-se que banhos terapêuticos com 500 mg/L de albendazol apresentaram alta eficácia (94,9%) contra monogenéticos de *C. macropomum*, causando poucas alterações fisiológicas e histopatológicas. Portanto, banhos com 500 mg/L de albendazol, na estratégia usada, podem ser recomendados para controle e tratamento de infecções por monogenéticos em *C*. *macropomum*.

## Introduction

Monogeneans are flatworm ectoparasites of marine and freshwater fish that can quickly infect entire stocks of fish because of their simple lifecycle and rapid transmission (Tavares-Dias & Martins, 2017; Costa et al., 2017). They cause excessive production of mucus on the skin and gills, along with hyperplasia, edema, fusion of secondary lamellae and gill necrosis (Alves et al., 2019; Tavares-Dias et al., 2021; Negreiros et al., 2022). This parasite is one of the main causes of problems within aquaculture worldwide. It can compromise the economic viability of production through negatively affecting growth, food conversion rates and the commercial value of fish (Soares et al., 2016; Alves et al., 2019; Hoai, 2020).

Because of the great importance of aquaculture around the world, enabling large-scale production of fish, it is the food production sector with the largest growth worldwide. Globally, aquaculture is responsible for greater fish production than are extractive fishing activities (Boyd et al., 2020; Tavares-Dias, 2021; Negreiros et al., 2022). Aquaculture is considered to be an important source of food, nutrition and income and a means of subsistence for hundreds of millions of people worldwide (FAO, 2018; Busatto et al., 2018; Hoai, 2020). However, these intensive production systems can provide environments that favor the emergence of parasitic diseases such as those caused by monogeneans (Quesada et al., 2013; Busatto et al., 2018; Hoai, 2020).

*Colossoma macropomum* is the most cultivated native species in Brazil and in almost the entire South American continent, as it has good zootechnical characteristics favorable to its intensive cultivation (Alves et al., 2019; Nogueira et al., 2019). However, with the growth of *C. macropomum* production, the emergence of parasitic diseases, mainly caused by monogeneans, has been inevitable (Tavares-Dias & Martins, 2017). Hence, adequate control and treatment for such diseases becomes necessary*.*

In aquaculture around the world, most of the chemotherapeutic agents used for disease control and treatment are chemical products derived from agriculture. In many countries, most of these products have never been regulated as specific medications for aquaculture (Busatto et al., 2018; Turnipseed et al., 2018). Many antiparasite drugs are used among animals of economic interest (Onaka et al., 2003). Among these drugs, albendazole has been studied in relation to treatment of parasitic diseases in fish.

Albendazole has been widely used within veterinary medicine (De Ruyck et al., 2000; Dar et al., 2017). This chemotherapeutic agent is the main component of benzimidazole, in the class of anthelmintic medications, which is used mainly for treating gastrointestinal parasitic infections in animals (Delatour & Parish, 1986; McKellar & Scott, 1990). Its action interrupts the energy metabolism of helminths (inhibition of fumarate reductase) or the polymerization of tubulin in the microtubules of these parasites (Manger, 1991; Dar et al., 2017). Because albendazole is widely used in fish farming worldwide, it has been studied in relation to control and treatment against monogeneans in different fish species (Buchmann & Bjerregaard, 1990; Tojo et al., 1992; Alves et al., 2019; Cordeiro et al., 2022; Negreiros et al., 2022).

Studies of fish blood parameters can be used as a prognostic indicator of pathological conditions, especially when considering changes in these parameters when fish are kept in different conditions. These assessments help to differentiate the response of fish when exposed to different substances (Ranzani-Paiva et al., 2013). Histopathological investigations allow identifying possible changes or tissue injuries, in addition to the severity, extent, evolution and intensity of alterations. Therefore, histology has been used as a tool to evaluate the effects of exposure to different chemical products on fish gills (Martins et al., 2018).

The aim of the present study was to investigate the antiparasite effects of therapeutic baths with albendazole and the hematological and histopathological alterations that these might cause in *C. macropomum*.

## Material and Methods

### Fish, acclimatization and the monogenean parasite

The fish used in this experiment were obtained from a commercial fish farm in Macapá, state of Amapá, Brazil. Juveniles of *C. macropomum* (104.0 ± 19.0 g) were transported to the laboratory where the trials were to be conducted. There, the fish were acclimatized over a seven-day period in a tank of capacity 500 L, constantly circulating water where they were fed twice a day with feed containing 32% crude protein. Any organic material that accumulated at the bottom of the tank was removed once a day. These fish, which were naturally parasitized by monogeneans, were used in all the trials.

### Anthelmintic chemical drugs

A solution of Albendazole (União Química Farmacêutica Nacional S/A, Brazil) was used at a concentration of 15% albendazole.

### *In vivo* assays *of C. macropomum* with albendazole

Specimens of *C. macropomum* (length 17.8 ± 1.0 cm and weight 109.0 ± 19.0 g) that were naturally parasitized by monogeneans were randomly distributed into six tanks of 100 L and were kept under an open water system for seven days for acclimatization. This trial consisted of two treatments with three replications each, containing 13 fish per replication. The treatments were as follows: a control group using tank water and a group subjected to baths using albendazole at 500 mg/L, a concentration that was determined through a previous study by Alves et al*.* (2019). The fish were maintained in a static water system from the time of addition of albendazole at this concentration until the end of a 24-hour period. The albendazole group was subjected to three of these 24-hour baths, with 24-hour intervals between the baths. During the treat ment period, the tank water was kept closed but with constant aeration.

After undergoing the three therapeutic baths, the fish were euthanized by sectioning the medulla. The gills of ten fish from each treatment were collected and fixed in 5% formalin, for quantification and identification of monogeneans, in accordance with the recommendations of Eiras et al. (2006). From the data obtained, the mean prevalence and abundance of infection were calculated (Bush et al., 1997). The efficacy of each treatment was then calculated in accordance with the methodology described previously by Zhang et al. (2014).

### Blood parameters of *C. macropomum* after the *in vivo* trial with albendazole

After the three therapeutic baths with albendazole at 500 mg/L, five fish from each replication (total of 15 fish per treatment) were used to evaluate blood parameters. A blood sample was collected from each fish by means of caudal vessel puncture using syringes containing EDTA (10%). Whole blood was used for the following determinations: hematocrit (Ht), through the microhematocrit method; total erythrocyte count (RBC), using a Neubauer chamber; and hemoglobin concentration (Hb), using the cyanmethemoglobin method. Wintrobe hematimetric indices such as mean corpuscular volume (MCV) and mean corpuscular hemoglobin concentration (MCHC) were calculated. Blood smears were made and stained panchromatically with the May-Grünwald-Giemsa-Wright combination, to make differential leukocyte counts in up to 200 cells of interest, in each blood smear. The determinations of total leukocyte and thrombocyte counts were done in accordance with previous recommendations (Ranzani-Paiva et al., 2013).

The remainder of the blood was centrifuged to obtain blood plasma. This was used to analyze plasma glucose levels by means of the glucose oxidase enzymatic-colorimetric method and to analyze total plasma proteins by means of the biuret method. Labtest kits were used in both of these analyses. The readings in both of these analyses were made using a Biospectro SP-220® spectrophotometer with different wavelengths.

### Histopathological analyses on the gills of *C. macropomum* after therapeutic baths with albendazole

After the three baths with albendazole, three fish from each replication (total of nine fish per treatment) were euthanized by sectioning the medulla. The first gill arch from each side (right and left) was collected for histopathological analysis. These samples were fixed directly in Davidson solution and were then dehydrated in an ascending series of ethyl alcohol. Following this, the material was embedded in paraffin, using routine techniques to prepare histological slides. Slides were produced in duplicate and were stained with hematoxylin and eosin (HE) for morphological analyses.

### Statistical analyses

The histopathological, parasitological and blood data were evaluated in relation to the assumptions of normal distribution using the Shapiro-Wilk test through the RVAideMemoire package (Herve, 2023) and homoscedasticity through the Car package (Fox & Weisberg, 2019). Because the data did not present normal distribution (p = 0.001), the Mann-Whitney test was used. These analyses were performed using the R Core Team version 4.2.2 statistical software (R Core Team, 2022).

## Results

### *In vivo* assays of albendazole against monogeneans in *C. macropomum*

Over the course of the three days of 24-hour therapeutic baths, there was no mortality among the fish, either in the group treated with albendazole at 500 mg/L on in the control group with tank water. The behavior of the fish was similar to what was described by Alves et al. (2019) during tolerance tests. All the specimens of *C. macropomum* used in the therapeutic baths presented gills parasitized by monogeneans (*Anacanthorus spathulatus*, *Notozothecium janauachensis* and *Mymarothecium boegeri*). There was a difference in the abundance of monogeneans between the treatment with albendazole at 500 mg/L and the control group (W = 868.5; p = 2.744e -10; p≤0,0001), such that there was a reduction in the parasite load in the treatment group, thus showing that the treatment had high efficacy (Table 1).

**Table 1:** Prevalence (P), mean abundance (MA) and efficacy of treatment (E) against monogeneans in the gills of *Colossoma macropomum* exposed to albendazole.

Treatments	P (%)	MA	E (%)
Water (control)	100	11.07 ± 11.55^a^	-
Albendazole at 500 mg/L	33.3	0.57 ± 1.01^b^	94.9

Different letters in the same column indicate significant differences between treatments (p<0.0001), according to the Mann-Whitney test.

### Blood parameters of *C. macropomum* exposed to albendazole

The fish exposed to albendazole at 500 mg/L presented increased plasma levels of glucose (W = 17, p = 0.0002158) and total protein (W = 39, p-value = 0.006822), compared with the fish in the control group, which were maintained in the cultivation tank water. The levels of hemoglobin (W = 62.5, p = 0.1073), hematocrit (W = 59, p = 0.07597), MCV (W = 87, p = 0.6347), MCHC (W = 97, p = 0.9817), number of total thrombocytes (W = 128, p = 0.1781), total leukocytes (W = 80, p = 0.4274), total lymphocytes (W = 79, p = 0.4013), total monocytes (W = 82, p = 0.4824) and total neutrophils (W = 66, p = 0.1499) remained unaltered by the baths with albendazole at 500 mg/L, compared with the control group exposed to cultivation tank water. The eosinophil numbers in the fish exposed to albendazole at 500 mg/L were decreased (W = 145, p = 0.03103), compared with the control (Table 2).

**Table 2:** Blood parameters of *Colossoma macropomum* exposed to albendazole at 500 mg/L.

Parameters	Water (control)	Albendazole at 500 mg/L
Glucose (g/dL)	64.4 ± 19.7^a^	107.3 ± 23.6^b^
Total protein (mg/dL)	2.8 ± 0.4^a^	3.2 ± 0.4^b^
Erythrocytes (x 10^6^ /μL)	1.43 ± 0.19^a^	1.48 ± 0.29^a^
Hemoglobin (g/dL	5.5 ± 0.5^a^	5.9 ± 0.8^a^
Hematocrit (%)	24.3 ± 2.0^a^	25.8 ± 3.1^a^
MCV (fL)	171.9 ± 20.1^a^	178.8 ± 29.9^a^
MCHC (g/dL)	22.9 ± 1.4^a^	23.0 ± 2.8^a^
Thrombocytes (μL)	88,023 ± 16,002^a^	77,691 ± 15,505^a^
Leukocytes (μL)	144,279 ± 21,156^a^	151,045 ± 30,756^a^
Lymphocytes (μL)	63,972 ± 10,103^a^	68,462 ± 15,341^a^
Monocytes (μL)	52,210 ± 6.290^a^	53,303 ± 9.393^a^
Neutrophils (μL)	16,109 ± 5.255^a^	19,260 ± 6.381^a^
Eosinophils (μL)	4.676 ± 1.567^a^	3.351 ± 1.810^b^
PAS-GL (μL)	6.451 ± 3.151^a^	6.032 ± 2.942^a^

Data express mean ± standard deviation. Different letters in the same line indicate differences according to the Mann-Whitney test (p≤0,0001). PAS-GL: Positive-PAS granular leukocytes; MCHC: Mean corpuscular hemoglobin concentration; MCV: Mean corpuscular volume.

### Histopathological analysis of the gills from *C. macropomum* exposed to albendazole

**Figure 1:**
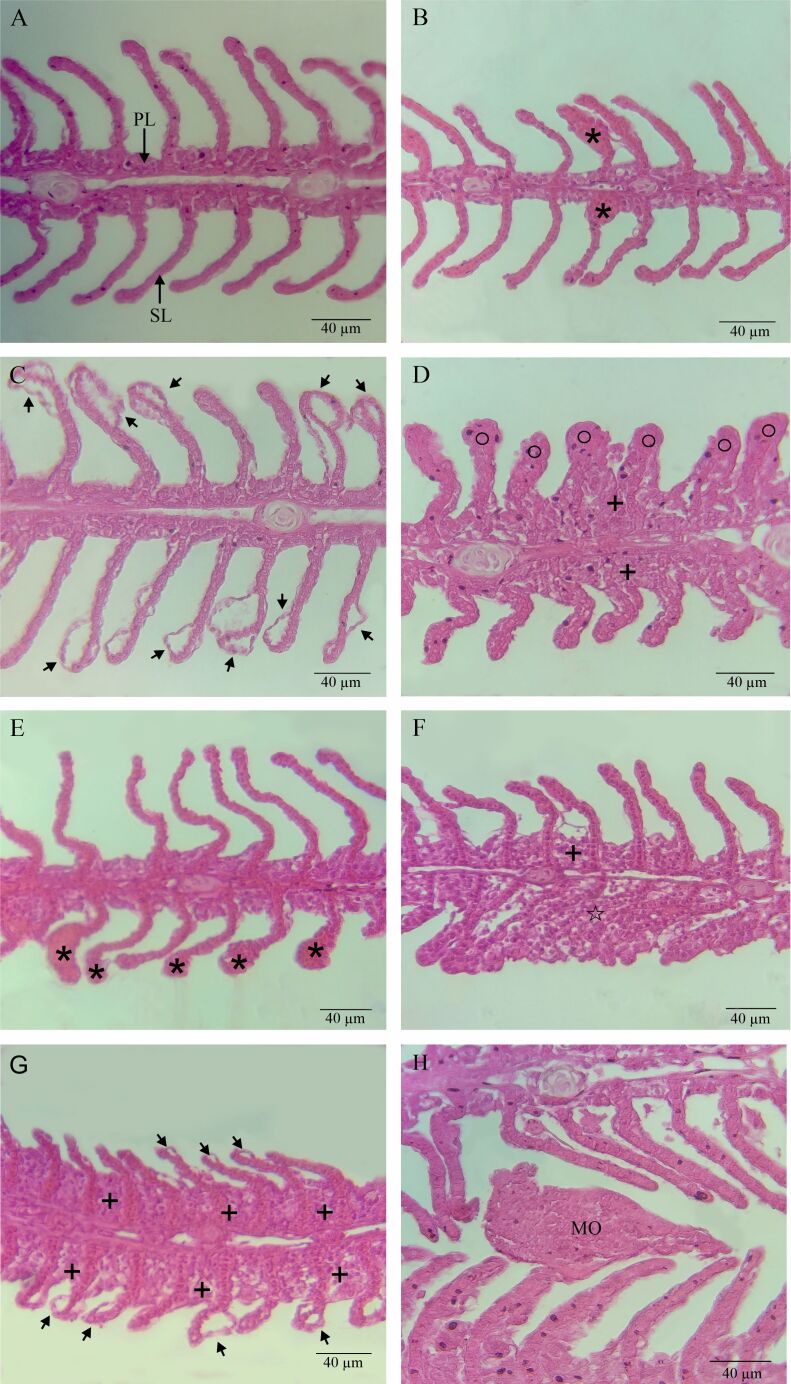
Histopathology of gills from *Colossoma macropomum* exposed to albendazole at 500 mg/L and cultivation tank water. (A) Gills of fish exposed to cultivation tank water (control) showing primary (PL) and secondary (SL) lamellae; (B) Aneurism (asterisk*) in the gills of fish exposed to cultivation tank water; (C) Detachment of the lamellar epithelium (arrow) in the gills of fish exposed to cultivation tank water; (D) Total hyperplasia (○) and hyperplasia with partial fusion of the lamellae (+) of fish exposed to cultivation tank water; (E) Aneurysm (*) in the gills of fish exposed to albendazole; (F) Total hyperplasia with fusion of the lamellae (☆) and hyperplasia with partial fusion of the lamellae (+) of fish exposed to albendazole; (G) Detachment of the lamellar epithelium (arrow) and hyperplasia with partial fusion of the lamellae in the gills (+) of fish exposed to albendazole; (H) Gill filament with monogenean (MO) in fish exposed to albendazole.

The fish exposed to albendazole at 500 mg/L and the control group with culture tank water presented histopathological alterations on the gills. These included detachment of epithelium, aneurysm and hyperplasia, which resulted in partial or total fusion of the secondary lamellae (Figure 1).

Comparative evaluation between the fish exposed to albendazole at 500 mg/L and the control group with cultivation tank water showed that there were no significant differences in the histological alteration index (HAI) (W = 37, p = 0.7855) or in the mean assessment values (MAV) (W = 29; p = 0.3263). Lesions with mild to moderate damage were recorded in both treatments (Table 3).

**Table 3:** Values of histopathological alteration index (HAI) and mean assessment values (MAV) for gills of *Colossoma macropomum* exposed to albendazole.

Treatments	N	MAV	HAI	Severity of the lesions according to HAI
Water	9	9.8 ± 1.9^a^	18.2 ± 55^a^	Mild to moderate organ damage
Albendazole at 500 mg/L	9	111 ± 2.9^a^	19.3 ± 4.8^a^	Mild to moderate organ damage

Data express mean ± standard deviation. Different letters in the same column indicate differences according to the Mann-Whitney test (p≤0,0001).

## Discussion

Monogenean infections are the most common diseases in different fish species, and these affect a large proportion of the world’s aquaculture production (Hoai, 2020). Infection by these helminths may result in considerable economic losses in aquaculture worldwide, but no estimates for the dimensions of these losses are yet available (Tavares-Dias & Martins, 2017).

One of the chemotherapeutic products used for controlling monogeneans in fish has been albendazole (Onaka et al., 2003; Alves et al., 2019; Negreiros et al., 2022). Three 24-hour baths with albendazole at 500 mg/L, with 24-hour intervals between them, gave rise to 94.9% efficacy against monogeneans of *C. macropomum* gills. Similarly, in a previous study (Negreiros et al., 2022), 24-hour bath with albendazole at 500 mg/L against monogeneans of *P. brachypomus* and *M. macrocephalus* had efficacies of 96.1% and 100%, respectively. However, in an evaluation of albendazole at 500 mg/L in a single 24-hour therapeutic bath against monogeneans of *C. macropomum*, Alves et al. (2019) found that its efficacy was only 48.6%. For *Piaractus mesopotamicus*, 30-minute baths with albendazole at 500 mg/L had efficacy of 56.9% against monogeneans, in an assessment after seven days of treatments (Onaka et al., 2003). Therefore, as expected, a larger number of baths with albendazole at 500 mg/L had greater efficacy against monogeneans of *C. macropomum* in the present study.

According to the Brazilian legislation on use of veterinary medications, these need to be registered with the Ministry of Agriculture, Livestock and Supply (MAPA) in order to be able to prescribe them for use within aquaculture. Moreover, to register a veterinary medication as an antiparasite agent in MAPA, its efficacy needs to be greater than or equal to 90% (Cordeiro et al., 2022). Therefore, the treatment with albendazole evaluated in the present study reaches this level of efficacy. However, studies evaluating the residual effects of albendazole on the muscles of fish and its clearance time remain necessary.

Synthetic drugs are used for controlling parasitosis caused by monogeneans in many different parts of the world and these present a range of levels of effectiveness and cause a number of side effects in the host fish. We observed that 24-hour baths with albendazole at 500 mg/L increased the levels of total plasma proteins and glucose in *C. macropomum*. The increase in glucose levels occurred because of the glycogenolytic and glyconeogenic effects of catecholamines and cortisol, respectively, and these increases have been used to measure responses to acute and chronic stress in fish (Barton & Iwama, 1991; Davis, 2006; Soares et al., 2016; Gonzales et al., 2020). Luz et al. (2021) stated that the total protein level depended on intracellular mechanisms and specific proteins that could be affected in stressed fish. Nevertheless, the other hematological parameters evaluated did not undergo any significant alterations, thus indicating that baths with albendazole at 500 mg/L caused few physiological effects in *C. macropomum*, possibly due to the 24-hour intervals between the treatments.

In the present study, exposure to albendazole at 500 mg/L caused structural alterations in the gills of *C. macropomum*. However, no lesions capable of impairing gill function were observed. The main histopathological alterations found, both in the control group with tank water and in the treatment with albendazole, were detachment of epithelium and hyperplasia resulting either in moderate or total fusion of the secondary lamellae. Other studies evaluating the use of albendazole at 500 mg/L in therapeutic baths also found hyperplasia of the epithelium coating the gills and caliciform cells in both, *P. mesopotamicus* exposed and in the control group. However, these alterations were more severe in the fish exposed to albendazole at 500 mg/L that presented the highest parasite loads (Onaka et al., 2003). It has been suggested that gill alterations such as detachment of epithelium and hyperplasia are adaptive and strategic actions for increasing the distance between the external environment and the blood, to make contact between blood and stressor agents more difficult (Winkaler et al., 2007). In the present study, although there was a histopathological difference in the abundance of monogeneans in the gills of *C. macropomum* between the treated and control groups, this was insufficient to cause differences between the treatments used.

In conclusion, three 24-hour therapeutic baths with albendazole at 500 mg/L presented high efficacy against monogeneans of *C. macropomum* gills, with few physiological or histopathological alterations. Therefore, the strategy of baths with albendazole at 500 mg/L used in the present study can be recommended for controlling and treating infections caused by monogeneans in this fish species.
